# SARS-CoV-2 Infection and Antibody Response in a Symptomatic Cat from Italy with Intestinal B-Cell Lymphoma

**DOI:** 10.3390/v13030527

**Published:** 2021-03-23

**Authors:** Julia Klaus, Carlo Palizzotto, Eric Zini, Marina L. Meli, Chiara Leo, Herman Egberink, Shan Zhao, Regina Hofmann-Lehmann

**Affiliations:** 1 Clinical Laboratory, Department of Clinical Diagnostics and Services, and Center for Clinical Studies, Vetsuisse Faculty, University of Zurich, Winterthurerstrasse 260, 8057 Zurich, Switzerland; mmeli@vetclinics.uzh.ch (M.L.M.); rhofmann@vetclinics.uzh.ch (R.H.-L.); 2 AniCura Istituto Veterinario Novara, Strada Provinciale 9, 28060 Granozzo con Monticello, Novara, Italy; ezini@vetclinics.uzh.ch (E.Z.); chiara.leo@anicura.it (C.L.); 3 Clinic for Small Animal Internal Medicine, Vetsuisse Faculty, University of Zurich, Winterthurerstrasse 260, 8057 Zurich, Switzerland; 4 Department of Animal Medicine, Production and Health, University of Padova, Viale dell’Università 16, 35020 Legnaro, Padova, Italy; 5 Department of Biomolecular Health Sciences, Faculty of Veterinary Medicine, University of Utrecht, 3584 CL Utrecht, The Netherlands; H.F.Egberink@uu.nl (H.E.); s.zhao@uu.nl (S.Z.)

**Keywords:** SARS-CoV-2, domestic cat, RT-qPCR, comorbidity, companion animal, B-cell lymphoma, human-to-feline transmission, serology, neutralizing activity

## Abstract

Since the coronavirus disease (COVID-19) pandemic was first identified in early 2020, rare cases of severe acute respiratory syndrome coronavirus-2 (SARS-CoV-2) infection in pet cats have been reported worldwide. Some reports of cats with SARS-CoV-2 showed self-limiting respiratory or gastrointestinal disease after suspected human-to-feline transmission via close contact with humans with SARS-CoV-2. In the present study, we investigated a cat with SARS-CoV-2 that was presented to a private animal clinic in Northern Italy in May 2020 in a weak clinical condition due to an underlying intestinal B-cell lymphoma. The cat developed signs of respiratory tract disease, including a sneeze, a cough and ocular discharge, three days after an oropharyngeal swab tested positive for SARS-CoV-2 viral RNA using two real-time reverse transcriptase polymerase chain reaction (RT-qPCR) assays for the envelope (*E*) and RNA-dependent RNA polymerase (*RdRp*) gene. Thus, SARS-CoV-2 viral RNA was detectable prior to the onset of clinical signs. Five and six months after positive molecular results, the serological testing substantiated the presence of a SARS-CoV-2 infection in the cat with the detection of anti-SARS-CoV-2 receptor binding domain (RBD) immunoglobulin (IgG) antibodies and neutralizing activity in a surrogate virus neutralization assay (sVNT). To the best of our knowledge, this extends the known duration of seropositivity of SARS-CoV-2 in a cat. Our study provides further evidence that cats are susceptible to SARS-CoV-2 under natural conditions and strengthens the assumption that comorbidities may play a role in the development of clinical disease.

## 1. Introduction

Severe acute respiratory syndrome coronavirus-2 (SARS-CoV-2) has spread worldwide among humans since the first cases were reported in January 2020 [1,2]. This novel coronavirus (CoV), which can lead to the coronavirus disease-19 (COVID-19) in humans, was presumably transmitted by animals, although the definitive animal origin has not yet been confirmed [3,4,5,6]. After this initial zoonotic event, the pandemic is now sustained by human-to-human transmission [7,8]. However, susceptibility has also been demonstrated in domestic and wild animals under natural and experimental conditions [9,10,11,12,13]. Studies showed that pet cats are able to transmit SARS-CoV-2 to cohoused cats after experimental virus inoculation [14,15,16], and in some reports, naturally infected cats developed clinical signs of respiratory and/or gastrointestinal disease [17,18,19,20,21]. Comorbidities such as hypertension, chronic kidney disease, diabetes mellitus and cancer were shown to increase the risk of the development of severe symptoms in humans with COVID-19 [22,23]. These morbidities are also known as the most common diseases in geriatric feline patients [24]. Pet cats are popular companion animals and often live in close contact with their owners. Especially in households affected by COVID-19, potential human-to-cat transmission and subsequent symptomatic SARS-CoV-2 infection need to be surveilled from a One-Health perspective.

Northern Italy was among the regions with the highest infection rates in the early phase of the pandemic. The daily confirmed cases of SARS-CoV-2 infection in humans peaked in Italy during the first wave at the end of March 2020, with around 6000 new cases per day [25]. At that time, six veterinary clinics from Northern Italy contributed to a SARS-CoV-2 surveillance study in collaboration with the Clinical Laboratory at the Vetsuisse Faculty Zurich, University of Zurich, Switzerland. For this study, oropharyngeal and nasal swabs from cats and dogs presented to the enrolled clinics were collected after the owners were informed and gave written consent. The study was approved by the local authorities and molecular, and subsequent serological testing was performed at the Clinical Laboratory, Zurich.

Here, we report the case of a cat with SARS-CoV-2, which was presented in weak clinical condition due to an underlying intestinal B-cell lymphoma. The cat developed moderate signs of respiratory disease three days after SARS-CoV-2 RNA was detected in RT-qPCR from an oropharyngeal swab. Anti-SARS-CoV-2 antibodies and neutralizing activity confirmed infection and were still present at five and six months after the positive RT-qPCR result. These findings support the urgent need for the surveillance of the role of cats in the pandemic and the evaluation of health risks posed by SARS-CoV-2 infections in cats, especially with underlying morbidities.

## 2. Case Report

**Figure 1:**
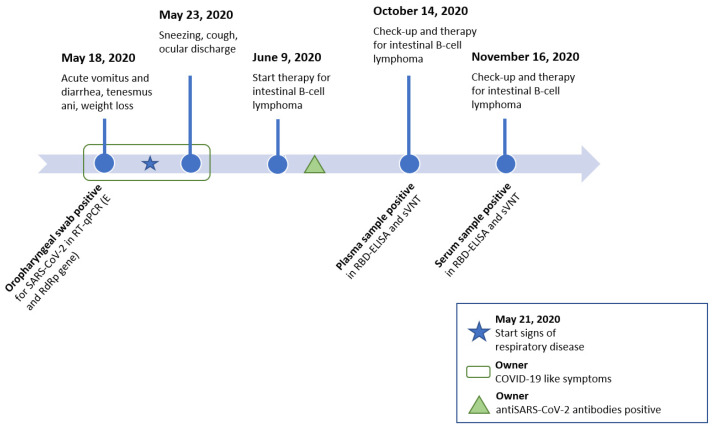
Timeline of consultations at the veterinary clinic (**top**) and sample collection for SARS-CoV-2 diagnostics including the results (**bottom**). Signs of respiratory disease (as indicated with the blue star) started three days after swab samples were collected. Abbreviations: real time reverse transcriptase polymerase chain reaction (RT-qPCR), envelope gene (*E* gene), RNA-dependent RNA polymerase gene (*RdRp* gene), receptor binding domain enzyme-linked immunosorbent assay (RBD-ELISA), surrogate virus neutralization test (sVNT).

A 12-year-old female spayed domestic short hair cat with no outdoor access was presented at a small animal clinic in the Piedmont region of Northern Italy on 18 May 2020 with signs described as acute vomitus and diarrhea, weakness, tenesmus ani and weight loss (Figure 1). The cat was already treated with the non-steroidal antiphlogistic drug (NSAID) meloxicam (Metacam, Boehringer Ingelheim Vetmedica GmbH, Ingelheim/Rhein, Germany) and the antibiotic cefovecin (Convenia, Zoetis, Parsippany, NJ, USA) by a private veterinarian three days prior, due to the described tenesmus. The cat was referred to the clinic after the onset of acute vomiting and diarrhea. The cat’s medical history indicated it had suffered a traumatic hip fracture eight years prior and a thymectomy, with the histologic diagnosis of thymoma recorded in 2018.

A clinical examination revealed that the cat weighed 3.9 kg and had a physiologic body condition score of 5/9, a heart murmur with a degree of II/VI, and tenesmus ani. No other abnormalities were found during the examination. For further assessment, complete blood work, urinalysis, and diagnostic imaging were performed. Ethylenediamine tetra-acetic acid (EDTA) anticoagulated blood for hematology and serum for biochemistry were collected. Hematology showed a moderate normocytic normochromic anemia (hematocrit 24.8%, reference range 28.2–52.7%, red blood cell count (RBC) 5.6 T/L, reference range 7.1–11.5 T/L). A hypoalbuminemia (19 g/L, reference range 27–44 g/L) and a mild hyperglycemia (176 mg/dL, reference range 63–140 mg/dL) were found in the biochemistry examination. All other measured biochemistry values were within the reference ranges.

On the same day, the cat was included in the multicentric study that aimed to evaluate the prevalence of SARS-CoV-2 in domestic cats and dogs. An oropharyngeal and a nasal swab were collected and stored in a prelabelled tube containing 300 µL of DNA/RNA shield solution (Zymo Research Europe GmbH, Freiburg, Germany). The swab samples were stored at 4 °C and shipped at ambient temperature to the clinical laboratory for further analysis.

Urinalysis was unremarkable, and thoracic radiographs showed an ill-defined area of increased opacity (approximately 2.5 cm × 1.5 cm × 1.2 cm) in the caudo-dorsal part of the right caudal lung lobe, which was already observed in 2018, and a diffuse bronchial wall thickening. The abdominal ultrasound identified splenomegaly with a honeycomb pattern and increased thickness of the intestinal muscular layer. A fine needle aspiration (FNA) of the spleen was performed, and a subsequent cytological assessment showed an increased number of lymphoid cells, a finding consistent with the differential diagnoses of lymphoid hyperplasia or lymphoma. The FNA specimen was sent for further analysis by polymerase chain reaction (PCR) for antigen receptor rearrangements (PARR), which later confirmed the presence of a monoclonal B-cell receptor arrangement (IDEXX Laboratories Inc., Westbrook, ME, USA). Based on the clinical findings and the PARR result, an intestinal B-cell lymphoma was then diagnosed.

On 23 May 2020, the cat was brought in again for consultation after having developed signs consistent with respiratory disease, especially sneezing, coughing and ocular discharge, two days earlier (Figure 1). The administration of aerosolized physiologic saline solution 0.9% was prescribed. At check-ups two and four days later, the cat continued to sneeze and showed inappetence. The results of hematology were unremarkable, and the clinical biochemistry revealed the persistence of hypoalbuminemia at 21 g/L (reference range: 27–44 g/L). However, the respiratory signs were self-limiting and resolved four days after the last check-up in May 2020. Additionally, at the same time as the cat showed signs of respiratory disease, the owner was suspected to have COVID-19. Later in summer 2020, the owner reported that a SARS-CoV-2 infection was confirmed by a positive SARS-CoV-2 antibody test. Additional information on the health status of the owner was not available. The cohoused cat had shown respiratory clinical signs comparable to the cat reported herein at the same time. However, samples from the cohoused cat and subsequent swab samples from the here reported cat were unavailable.

Chemotherapy with lomustine 50 mg/m^2^ and prednisolone 2 mg/kg began on 9 June 2020. After two weeks, hematologic analysis revealed a neutropenia grade 2 (1162 cells/µL, reference range: 2620–15170 cells/µL). However, the cat was in good clinical condition, its body weight had increased, and no clinical signs compatible with infectious disease were observed. Twenty-eight days after the previous administration, the cat was in good condition, lomustine was administered at the same dosage and treatment continued every 28 days, except prednisolone administration was stopped after the first month.

As part of the SARS-CoV-2 surveillance study in companion animals, the oropharyngeal and nasal swabs were analyzed in Zurich on 28 September 2020 using reverse-transcriptase real-time polymerase chain reaction (RT-qPCR) as previously described [26].

Briefly, the total nucleic acids (TNA) were extracted from both swabs using a MagNA Pure LC 2.0 instrument (Roche Diagnostics AG, Rotkreuz, Switzerland) with the MagNA Pure LC Total Nucleic Acid—High Performance Kit (Roche Diagnostics AG). Subsequently, 4 µL of the sample TNA was used for a RT-qPCR with 20 µL of total reaction volume. The reaction included a TaqMan^®^ Fast Virus 1-Step Master Mix (Applied Biosystems, Foster City, CA, USA) and targeted the envelope (E) or RNA-dependent RNA polymerase (RdRp) gene. The oropharyngeal swab of the cat yielded RT-qPCR-positive results with an amplification in the E and RdRp genes of SARS-CoV-2. The cycle threshold (CT) values were high, with 39 in the *E* gene and 41 in the *RdRp* gene, and were, therefore, interpreted as questionable positive [26]. The nasal swab tested negative for SARS-CoV-2 RNA. The result from the oropharyngeal swab was confirmed as positive by the Swiss Federal Institute for Virology and Immunology (IVI, Mittelhäusern, Switzerland), with CT values ranging from 36.6 to 37.2 in RT-qPCR assays targeting the E and RdRp genes of SARS-CoV-2 [27]. Results with a CT value ≤38 are considered positive. Both swabs were analyzed for the presence of feline calicivirus (FCV) and feline herpesvirus (FHV) nucleic acids by RT-qPCR and qPCR [28,29], respectively, yielding negative results.

**Figure 2:**
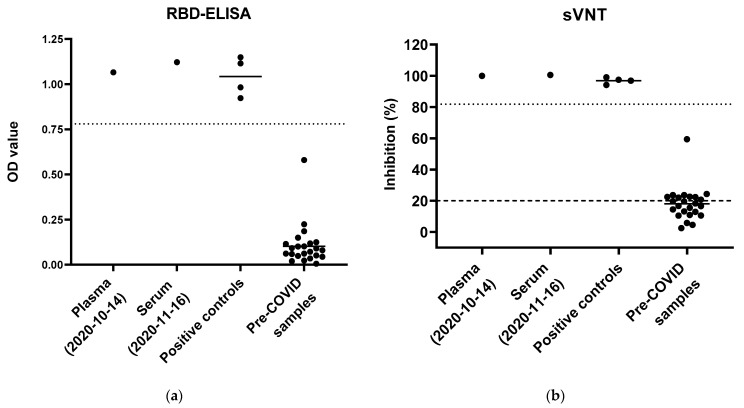
Serological results from blood collected in October and November 2020. Positive controls (*n* = 4) and pre-COVID sera (*n* = 24, collected before 5 January 2020) were run in parallel in both assays. (**a**) Enzyme-linked immunosorbent assay (ELISA) for the detection of anti-SARS-CoV-2 spike protein receptor binding domain (RBD) antibodies. Results are presented by optical density values (OD) and were measured at 415 nm. The mean OD is shown as a line within the group. The dotted line shows the positive cut-off value set at 0.78. (**b**) A surrogate virus neutralization test (sVNT) was used to measure neutralizing activity. The dashed line indicates the positive cut-off set for human samples by the manufacturer at ≥20%, and the dotted line indicates the positive cut-off calculated at 81.8%.

Thereafter, the cat was presented to the clinic for the monthly application of chemotherapy (14 October 2020 and 16 November 2020) and was in good clinical condition. The hematology and biochemistry were unremarkable. The remains of the collected blood (EDTA plasma and serum) were sent for serological testing to the clinical laboratory in Switzerland. Two serological tests were performed for the detection of anti-SARS-CoV-2 antibodies. An in-house established enzyme-linked immunosorbent assay (ELISA), which detects immunoglobulins G (IgG) against the receptor binding domain (RBD) of the viral spike protein S1 domain, was conducted with the patient’s EDTA plasma and serum as previously described [26]. Briefly, a 96-well MICROLON^®^, C-bottom, medium binding plate (Greiner-Bio One, St. Gallen, Switzerland) was coated with 200 ng/well of recombinant spike glycoprotein RBD SARS-related Coronavirus 2, Wuhan-Hu-1 with C-Terminal Histidine Tag (NR-52946, BEI Resources, Manassas, United States). Samples and controls were heat inactivated and diluted at 1:100 with P3x buffer, which contains 0.15 M sodium chloride, 1 mM Na_2_-EDTA (Titriplex^®^ III, VWR, Dietikon, Switzerland), 0.05 M Tris-base (tris(hydroxymethyl)aminomethane, Fisher Scientific, Rheinach, Switzerland), 0.1% bovine serum albumin (BSA) and 0.1% Tween 20. For each reaction, 100 µL of diluted samples or controls was used and pipetted in duplicate. Twenty-four pre-COVID sera from Swiss cats collected before January 2020 and four positive control sera were run in parallel as previously described [26]. The positive control sera were provided by Dr Herman Egberink and Dr Els Broens, Faculty of Veterinary Medicine, University of Utrecht, the Netherlands. The plasma sample from 14 October 2020 and the serum sample from 16 November 2020 yielded positive results in the ELISA with mean optical density (OD) values of 1.07 and 1.12, respectively (Figure 2a). For this assay, the positive cut-off was set at six-fold standard deviations above the mean OD of pre-COVID sera at 0.78 [26,30].

The neutralizing activity in the plasma was measured with the commercially available surrogate virus neutralization test kit (sVNT, GenScript Inc., Piscataway, NJ, USA). In this test, the inhibition of the binding of the viral spike glycoprotein RBD to an angiotensin converting enzyme-2 (ACE2) cell surface receptor is assessed independent of species identity. The test was conducted according to the manufacturer’s instructions. Negative and positive controls provided by the kit were run in parallel and the measured OD values ranged within the required references to assure test validity. Together with the samples, 24 pre-COVID sera and four positive control sera were analyzed. According to the manufacturer’s directions, an inhibition of ≥20% is stated as positive for human samples. As reported earlier, we calculated a positive cut-off as six-fold standard deviation above the mean inhibition of the pre-COVID sera, which was set at 81.8% [26]. The cat’s plasma from 14 October 2020 and the serum from 16 November 2020 both yielded positive inhibition measurements of 99.9 and 100.5%, respectively (Figure 2b).

For confirmation of the detected antibody response, the plasma from 14 October 2020 was sent to the Virology Division of the Faculty of Veterinary Medicine, University of Utrecht where seropositivity was verified with a virus neutralization assay with vesicular stomatitis virus (VSV) particles pseudotyped with SARS-CoV-2 spike protein [31,32] and yielded a titer of 1:256 (a titer ≥16 is considered positive).

The serum sample from 16 November 2020 was also tested for feline immunodeficiency virus (FIV) using Western blot and feline leukemia virus (FeLV) p27 antigen by sandwich ELISA as previously described [33,34,35,36]. Both tests yielded negative results. No samples were available from the cohoused cat.

## 3. Discussion

We hereby report the case of a 12-year-old female spayed domestic shorthair cat with SARS-CoV-2, which developed moderate signs of respiratory disease, while exhibiting a weak clinical condition due to an underlying intestinal B-cell lymphoma. An oropharyngeal swab, collected shortly before the cat developed signs of respiratory disease, yielded positive results for SARS-CoV-2 in RT-qPCR in the *E* and *RdRp* gene. The measured CT-values, which ranged from 36.6 to 41 depending on the performed assay and targeted gene, reflected low viral RNA loads. These low viral RNA loads may be explained by the early timepoint of sampling, three days before the onset of signs of respiratory disease. Sequencing of the viral RNA was not attempted due to the low viral RNA loads, and no subsequent swab samples were available for further investigation of viral RNA shedding. The swab samples tested negative for common feline respiratory viral agents (FCV and FHV) and no evidence of FIV or progressive FeLV infection was detected in the cat. Given that other common feline viral infections were excluded, we assume that the SARS-CoV-2 infection played a role in the development of respiratory disease in the cat. At the same time, the cat owner experienced symptoms compatible with SARS-CoV-2 infection, which was later confirmed by a serological test. In May 2020, the daily confirmed new SARS-CoV-2-positive cases for humans in Italy had declined: in the region of Piedmont, there were 9874 total active cases and 72 new cases noted on 18 May 2020 [37].

Six months after the SARS-CoV-2 viral RNA-positive oropharyngeal swab was collected, serological tests confirmed SARS-CoV-2 infection in the cat by the detection of anti-SARS-CoV-2 spike protein RBD-antibodies and neutralizing activity in an ELISA-based surrogate virus neutralization test. Between the fifth and sixth month, the antibody response remained unchanged. This further strengthens the assumption that the cat had become infected at the timepoint of the detection of viral RNA and the onset of respiratory signs and antibodies to SARS-CoV-2 were still present six months later. This seems to be the longest time span to date that the immune response to SARS-CoV-2 infection in a pet cat was detectable. A recent report demonstrated that antibodies in naturally infected pet cats persist as long as two months [38]. A large-scale surveillance study, which was conducted from March to May 2020 with samples collected mostly in Lombardy, a neighboring region of Piedmont, reported no PCR-positive cats (0/180, including 57 cats from previously confirmed households affected by COVID-19 and 38 cats with respiratory signs), but detected neutralizing antibodies in 5.8% (11/191) of the tested cats [39].

Since the pandemic was first identified in 2020, small numbers of SARS-CoV-2 infections in cats under natural conditions have been reported. In almost all of these cases, human-to-animal transmission was suspected, and to date, no zoonotic events involving infected cats have been described [13]. However, proof of cat-to-human SARS-CoV-2 transmission will unlikely be obtainable, for ethical and study design considerations [40]. By contrast, animal-to-human transmission was shown at infected mink farms [11,41,42,43]. In cats, clinical disease accompanied with natural SARS-CoV-2 infection could be observed in some cases, with cats showing respiratory and/or gastrointestinal signs [13,20,44,45]. Notably, most cats that were infected under experimental conditions did not develop clinical signs, although viral shedding was present from day one up to day seven post infection [14,15,16,46]. However, only cats without underlying health impairments were included in experimental studies, unlike the pet cat population presented to veterinarians. Human patients with underlying clinical conditions or immunocompromised humans were shown to have a higher risk of developing severe clinical disease when infected with SARS-CoV-2 [22,23], and a previous report from Spain in felines suspected the contribution of comorbidities to the clinical outcome in a cat that was found to be SARS-CoV-2 RT-qPCR positive using nasal swabs while suffering from severe respiratory distress and thrombocytopenia. After the cat was euthanized and a necropsy had been conducted, the cat was diagnosed with feline hypertrophic cardiomyopathy, severe pulmonary edema and thrombosis [18]. Here, we described the case of a cat with symptomatic SARS-CoV-2 infection while suffering from intestinal B-cell lymphoma.

In many countries, the molecular detection of SARS-CoV-2 in pets is currently limited to research studies or, in the case of diagnostic purposes, is restricted to specific regulations, such as contact with humans with SARS-CoV-2 and the development of clinical disease potentially related to SARS-CoV-2 infection. Therefore, the prevalence of SARS-CoV-2 infection in cats, especially asymptomatic cases, may be underestimated [38]. However, SARS-CoV-2 diagnostics in cats living in critical environments, such as residencies for elderly people, dense housing conditions and cat rescue and breeding facilities, are of importance. Furthermore, the recently emerged variants (B.1.1.7. and B.1.351) may have a fitness advantage associated with mutations in the spike protein, which are suspected to lead to an increase in human-to-human transmissibility and more effective replication [47,48]. Possible changes in the susceptibility of animals, in the context of these new variants, should be evaluated.

As part of infection prevention, humans with SARS-CoV-2 should limit close contact with animals and apply strict hygiene measures, similar to those implemented when in contact with uninfected humans [49]. Such hygiene measures for pet owners include “handling animals only when wearing a mask, washing their hands with soap and water for at least 20 s before and after being near or handling their animals, their food, or their supplies, as well as avoiding kissing their pets or sharing food, towels, or the bed with them”, as recommended by the European Advisory Board on Cat Diseases (ABCD) [50,51].

This novel viral infection has proven its potential for interspecies transmission and zoonosis. A SARS-CoV-2 infection presents a health risk to cats, which may be increased by comorbidities. Thus, SARS-CoV-2 needs to be closely surveilled in the human–animal interface.

## 4. Conclusions

The current case adds important information to the field, as SARS-CoV-2 infection was detected in a cat in poor condition due to an underlying intestinal B-cell lymphoma and three days before the onset of respiratory signs. Seropositivity was present for at least half a year after the oropharyngeal swab from this cat tested positive for SARS-CoV-2 RNA. Further investigations in susceptibility, immunity and the role of comorbidities as promotive factors for SARS-CoV-2-related disease in cats are essential.

## Data Availability

All available data are presented in this manuscript.

## References

[1] Coronaviridae Study Group of the International Committee on Taxonomy of Viruses (2020). The species Severe acute respiratory syndrome-related coronavirus: Classifying 2019-nCoV and naming it SARS-CoV-2. Nat. Microbiol..

[2] Zhou P., Yang X.-L., Wang X.-G., Hu B., Zhang L., Zhang W., Si H.-R., Zhu Y., Li B., Huang C.-L. (2020). A pneumonia outbreak associated with a new coronavirus of probable bat origin. Nature.

[3] Andersen K.G., Rambaut A., Lipkin W.I., Holmes E.C., Garry R.F. (2020). The proximal origin of SARS-CoV-2. Nat. Med..

[4] Lu R., Zhao X., Li J., Niu P., Yang B., Wu H., Wang W., Song H., Huang B., Zhu N. (2020). Genomic characterisation and epidemiology of 2019 novel coronavirus: Implications for virus origins and receptor binding. Lancet.

[5] Wang H., Li X., Li T., Zhang S., Wang L., Wu X., Liu J. (2020). The genetic sequence, origin, and diagnosis of SARS-CoV-2. Eur. J. Clin. Microbiol. Infect Dis..

[6] Zhang T., Wu Q., Zhang Z. (2020). Probable Pangolin Origin of SARS-CoV-2 Associated with the COVID-19 Outbreak. Curr. Biol..

[7] World Health Organization Transmission of SARS-CoV-2: Implications for Infection Prevention Precautions. https://www.who.int/news-room/commentaries/detail/transmission-of-sars-cov-2-implications-for-infection-prevention-precautions.

[8] Chan J.F., Yuan S., Kok K.H., To K.K., Chu H., Yang J., Xing F., Liu J., Yip C.C., Poon R.W. (2020). A familial cluster of pneumonia associated with the 2019 novel coronavirus indicating person-to-person transmission: A study of a family cluster. Lancet.

[9] McAloose D., Laverack M., Wang L., Killian M.L., Caserta L.C., Yuan F., Mitchell P.K., Queen K., Mauldin M.R., Cronk B.D. (2020). From People to Panthera: Natural SARS-CoV-2 Infection in Tigers and Lions at the Bronx Zoo. mBio.

[10] Wang L., Mitchell P.K., Calle P.P., Bartlett S.L., McAloose D., Killian M.L., Yuan F., Fang Y., Goodman L.B., Fredrickson R. (2020). Complete Genome Sequence of SARS-CoV-2 in a Tiger from a U.S. Zoological Collection. Microbiol. Resour. Announc..

[11] Oreshkova N., Molenaar R.J., Vreman S., Harders F., Oude Munnink B.B., Hakze-van der Honing R.W., Gerhards N., Tolsma P., Bouwstra R., Sikkema R.S. (2020). SARS-CoV-2 infection in farmed minks, the Netherlands, April and May 2020. Euro Surveill..

[12] Sit T.H.C., Brackman C.J., Ip S.M., Tam K.W.S., Law P.Y.T., To E.M.W., Yu V.Y.T., Sims L.D., Tsang D.N.C., Chu D.K.W. (2020). Infection of dogs with SARS-CoV-2. Nature.

[13] OIE—World Organization for Animal Health Events in Animals. https://www.oie.int/en/scientific-expertise/specific-information-and-recommendations/questions-and-answers-on-2019novel-coronavirus/events-in-animals/.

[14] Bosco-Lauth A.M., Hartwig A.E., Porter S.M., Gordy P.W., Nehring M., Byas A.D., VandeWoude S., Ragan I.K., Maison R.M., Bowen R.A. (2020). Experimental infection of domestic dogs and cats with SARS-CoV-2: Pathogenesis, transmission, and response to reexposure in cats. Proc. Natl. Acad. Sci. USA.

[15] Halfmann P.J., Hatta M., Chiba S., Maemura T., Fan S., Takeda M., Kinoshita N., Hattori S.I., Sakai-Tagawa Y., Iwatsuki-Horimoto K. (2020). Transmission of SARS-CoV-2 in Domestic Cats. N. Engl. J. Med..

[16] Shi J., Wen Z., Zhong G., Yang H., Wang C., Huang B., Liu R., He X., Shuai L., Sun Z. (2020). Susceptibility of ferrets, cats, dogs, and other domesticated animals to SARS-coronavirus 2. Science.

[17] Newman A., Smith D., Ghai R.R., Wallace R.M., Torchetti M.K., Loiacono C., Murrell L.S., Carpenter A., Moroff S., Rooney J.A. (2020). First Reported Cases of SARS-CoV-2 Infection in Companion Animals—New York, March-April 2020. Mmwr Morb. Mortal. Wkly. Rep..

[18] Segalés J., Puig M., Rodon J., Avila-Nieto C., Carrillo J., Cantero G., Terrón M.T., Cruz S., Parera M., Noguera-Julián M. (2020). Detection of SARS-CoV-2 in a cat owned by a COVID-19-affected patient in Spain. Proc. Natl. Acad. Sci. USA.

[19] Garigliany M., Van Laere A.-S., Clercx C., Giet D., Escriou N., Huon C., van der Werf S., Eloit M., Desmecht D. (2020). SARS-CoV-2 Natural Transmission from Human to Cat, Belgium, March 2020. Emerg. Infect. Dis. J..

[20] Sailleau C., Dumarest M., Vanhomwegen J., Delaplace M., Caro V., Kwasiborski A., Hourdel V., Chevaillier P., Barbarino A., Comtet L. (2020). First detection and genome sequencing of SARS-CoV-2 in an infected cat in France. Transbound. Emerg. Dis..

[21] Hosie M.J., Epifano I., Herder V., Orton R.J., Stevenson A., Johnson N., MacDonald E., Dunbar D., McDonald M., Howie F. (2020). Respiratory disease in cats associated with human-to-cat transmission of SARS-CoV-2 in the UK. bioRxiv.

[22] Ng W.H., Tipih T., Makoah N.A., Vermeulen J.-G., Goedhals D., Sempa J.B., Burt F.J., Taylor A., Mahalingam S. (2021). Comorbidities in SARS-CoV-2 Patients: A Systematic Review and Meta-Analysis. mBio.

[23] Yang J., Zheng Y., Gou X., Pu K., Chen Z., Guo Q., Ji R., Wang H., Wang Y., Zhou Y. (2020). Prevalence of comorbidities and its effects in patients infected with SARS-CoV-2: A systematic review and meta-analysis. Int. J. Infect. Dis..

[24] Bellows J., Center S., Daristotle L., Estrada A.H., Flickinger E.A., Horwitz D.F., Lascelles B.D.X., Lepine A., Perea S., Scherk M. (2016). Aging in cats:Common physical and functional changes. J. Feline Med. Surg..

[25] John Hopkins University COVID-19 Dashboard by the Center for Systems Science and Engineering (CSSE) at Johns Hopkins University (JHU). https://coronavirus.jhu.edu/map.html.

[26] Klaus J., Meli M.L., Willi B., Nadeau S., Beisel C., Stadler T., Egberink H., Zhao S., Lutz H., Riond B. (2021). ETH SARS-CoV-2 Sequencing Team. Detection and Genome Sequencing of SARS-CoV-2 in a Domestic Cat with Respiratory Signs in Switzerland. Viruses.

[27] Corman V.M., Landt O., Kaiser M., Molenkamp R., Meijer A., Chu D.K., Bleicker T., Brünink S., Schneider J., Schmidt M.L. (2020). Detection of 2019 novel coronavirus (2019-nCoV) by real-time RT-PCR. Euro Surveill..

[28] Berger A., Willi B., Meli M.L., Boretti F.S., Hartnack S., Dreyfus A., Lutz H., Hofmann-Lehmann R. (2015). Feline calicivirus and other respiratory pathogens in cats with Feline calicivirus-related symptoms and in clinically healthy cats in Switzerland. BMC Vet. Res..

[29] Vögtlin A., Fraefel C., Albini S., Leutenegger C.M., Schraner E., Spiess B., Lutz H., Ackermann M. (2002). Quantification of Feline Herpesvirus 1 DNA in Ocular Fluid Samples of Clinically Diseased Cats by Real-Time TaqMan PCR. J. Clin. Microbiol..

[30] Okba N.M.A., Müller M., Li W., Wang C., GeurtsvanKessel C., Corman V., Lamers M., Sikkema R., de Bruin E., Chandler F. (2020). Severe Acute Respiratory Syndrome Coronavirus 2−Specific Antibody Responses in Coronavirus Disease Patients. Emerg. Infect. Dis. J..

[31] Wang C., Li W., Drabek D., Okba N.M.A., van Haperen R., Osterhaus A.D.M.E., van Kuppeveld F.J.M., Haagmans B.L., Grosveld F., Bosch B.-J. (2020). A human monoclonal antibody blocking SARS-CoV-2 infection. Nat. Commun..

[32] Zhao S., Schuurman N., Li W., Wang C., Smit L.A., Broens E.M., Wagenaar J.A., van Kuppeveld F.J., Bosch B.J., Egberink H. (2021). Serologic screening of severe acute respiratory syndrome coronavirus 2 infection in cats and dogs during first coronavirus disease wave, the Netherlands. Emerg. Infect. Dis..

[33] Egberink H.F., Lutz H., Horzinek M.C. (1991). Use of western blot and radioimmunoprecipitation for diagnosis of feline leukemia and feline immunodeficiency virus infections. J. Am. Vet. Med. Assoc..

[34] Lutz H., Pedersen N.C., Durbin R., Theilen G.H. (1983). Monoclonal antibodies to three epitopic regions of feline leukemia virus p27 and their use in enzyme-linked immunosorbent assay of p27. J. Immunol. Methods.

[35] Frankenfeld J., Meili T., Meli M.L., Riond B., Helfer-Hungerbuehler A.K., Bönzli E., Pineroli B., Hofmann-Lehmann R. (2019). Decreased Sensitivity of the Serological Detection of Feline Immunodeficiency Virus Infection Potentially Due to Imported Genetic Variants. Viruses.

[36] Calzolari M., Young E., Cox D., Davis D., Lutz H. (1995). Serological diagnosis of feline immunodeficiency virus infection using recombinant transmembrane glycoprotein. Vet. Immunol. Immunopathol..

[37] La Repubblica Coronavirus, il bollettino del 18 maggio: I 99 morti e meno di 500 nuovi contagiati. https://www.repubblica.it/cronaca/2020/05/18/news/coronavirus_bollettino_18_maggio-257012259/.

[38] Hamer S.A., Pauvolid-Corrêa A., Zecca I.B., Davila E., Auckland L.D., Roundy C.M., Tang W., Torchetti M., Killian M.L., Jenkins-Moore M. (2020). Natural SARS-CoV-2 infections, including virus isolation, among serially tested cats and dogs in households with confirmed human COVID-19 cases in Texas, USA. bioRxiv.

[39] Patterson E.I., Elia G., Grassi A., Giordano A., Desario C., Medardo M., Smith S.L., Anderson E.R., Prince T., Patterson G.T. (2020). Evidence of exposure to SARS-CoV-2 in cats and dogs from households in Italy. Nat. Commun..

[40] Totton S.C., Sargeant J.M., O’Connor A.M. (2020). How could we conclude cat-to-human transmission of SARS-CoV-2?. Zoonoses Public Health.

[41] Oude Munnink B.B., Sikkema R.S., Nieuwenhuijse D.F., Molenaar R.J., Munger E., Molenkamp R., van der Spek A., Tolsma P., Rietveld A., Brouwer M. (2021). Transmission of SARS-CoV-2 on mink farms between humans and mink and back to humans. Science.

[42] World Health Organization SARS-CoV-2 Mink-Associated Variant Strain—Denmark. https://www.who.int/csr/don/06-november-2020-mink-associated-sars-cov2-denmark/en/.

[43] Larsen H.D., Fonager J., Lomholt F.K., Dalby T., Benedetti G., Kristensen B., Urth T.R., Rasmussen M., Lassaunière R., Rasmussen T.B. (2021). Preliminary report of an outbreak of SARS-CoV-2 in mink and mink farmers associated with community spread, Denmark, June to November 2020. Euro Surveill..

[44] de Morais H.A., Dos Santos A.P., do Nascimento N.C., Kmetiuk L.B., Barbosa D.S., Brandão P.E., Guimarães A.M.S., Pettan-Brewer C., Biondo A.W. (2020). Natural Infection by SARS-CoV-2 in Companion Animals: A Review of Case Reports and Current Evidence of Their Role in the Epidemiology of COVID-19. Front. Vet. Sci..

[45] Centers for Disease Control and Prevention CDC Confirmation of COVID-19 in Two Pet Cats in New York. https://www.cdc.gov/media/releases/2020/s0422-covid-19-cats-NYC.html.

[46] Gaudreault N.N., Trujillo J.D., Carossino M., Meekins D.A., Morozov I., Madden D.W., Indran S.V., Bold D., Balaraman V., Kwon T. (2020). SARS-CoV-2 infection, disease and transmission in domestic cats. Emerg. Microbes Infect..

[47] Volz E., Hill V., McCrone J.T., Price A., Jorgensen D., O’Toole Á., Southgate J., Johnson R., Jackson B., Nascimento F.F. (2021). Evaluating the Effects of SARS-CoV-2 Spike Mutation D614G on Transmissibility and Pathogenicity. Cell.

[48] Zhou B., Thao T.T.N., Hoffmann D., Taddeo A., Ebert N., Labroussaa F., Pohlmann A., King J., Portmann J., Halwe N.J. (2020). SARS-CoV-2 spike D614G variant confers enhanced replication and transmissibility. bioRxiv.

[49] Centers for Disease Control and Prevention CDC COVID-19, If You Have Pets. https://www.cdc.gov/coronavirus/2019-ncov/daily-life-coping/pets.html.

[50] Hosie M.J., Hofmann-Lehmann R., Hartmann K., Egberink H., Truyen U., Addie D.D., Belák S., Boucraut-Baralon C., Frymus T., Lloret A. (2021). Anthropogenic Infection of Cats during the 2020 COVID-19 Pandemic. Viruses.

[51] European Advisory Board on Cat Diseases (ABCD) ABCD Guidelines SARS-CoV-2 in Cats. http://www.abcdcatsvets.org/sars-coronavirus-2-and-cats/.

